# Supplementary material for: High-resolution deconstruction of evolution induced by chemotherapy treatments in breast cancer xenografts
Source: Sci Rep. 2018 Dec 18;8:17937. doi: 10.1038/s41598-018-36184-8 (PMC6298990; doi:10.1038/s41598-018-36184-8)
Supplement: Supplementary file 1 — Supplementary Figures [file 41598_2018_36184_MOESM1_ESM.pdf]

Supplementary materials

High-resolution deconstruction of evolution induced by chemotherapy treatments in breast cancer xenografts

Authors: Hyunsoo Kim[1], Pooja Kumar[1], Francesca Menghi[1], Javad Noorbakhsh[1], Eliza Cerveira[1], Mallory Ryan[1], Qihui Zhu[1], Guruprasad Ananda[1], Joshy George[1], Henry C. Chen[2], Susan Mockus[1], Chengsheng Zhang[1], Yan Yang[2], James Keck[2], R. Krishna Murthy Karuturi[3], Carol J. Bult[3], Charles Lee[1], Edison T. Liu[3], Jeffrey H. Chuang[1,4,*]

Affiliations: [1]The Jackson Laboratory for Genomic Medicine, Farmington, CT 06030, USA. [2]In Vivo Services, JAX® Mice, Clinical & Research Services, The Jackson Laboratory, Sacramento, CA 95838, USA. [3]The Jackson Laboratory, Bar Harbor, ME 04609, USA. [4]UConn Health, Department of Genetics and Genome Sciences. Farmington, CT 06030, USA. [*]Corresponding author.

Corresponding Author: Jeffrey Chuang. The Jackson Laboratory for Genomic Medicine, 10 Discovery Drive, Farmington CT 06032. 860-837-2473. jeff.chuang@jax.org

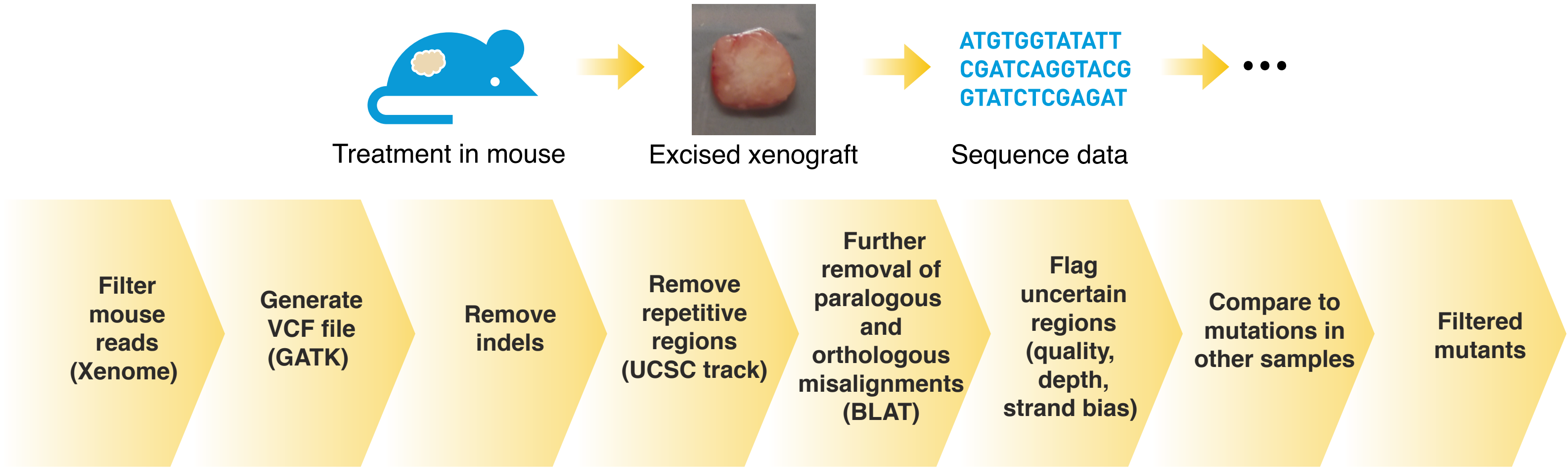

Figure S1. A high specificity mutation-calling pipeline for comparative evolutionary analysis across exome-seq samples.

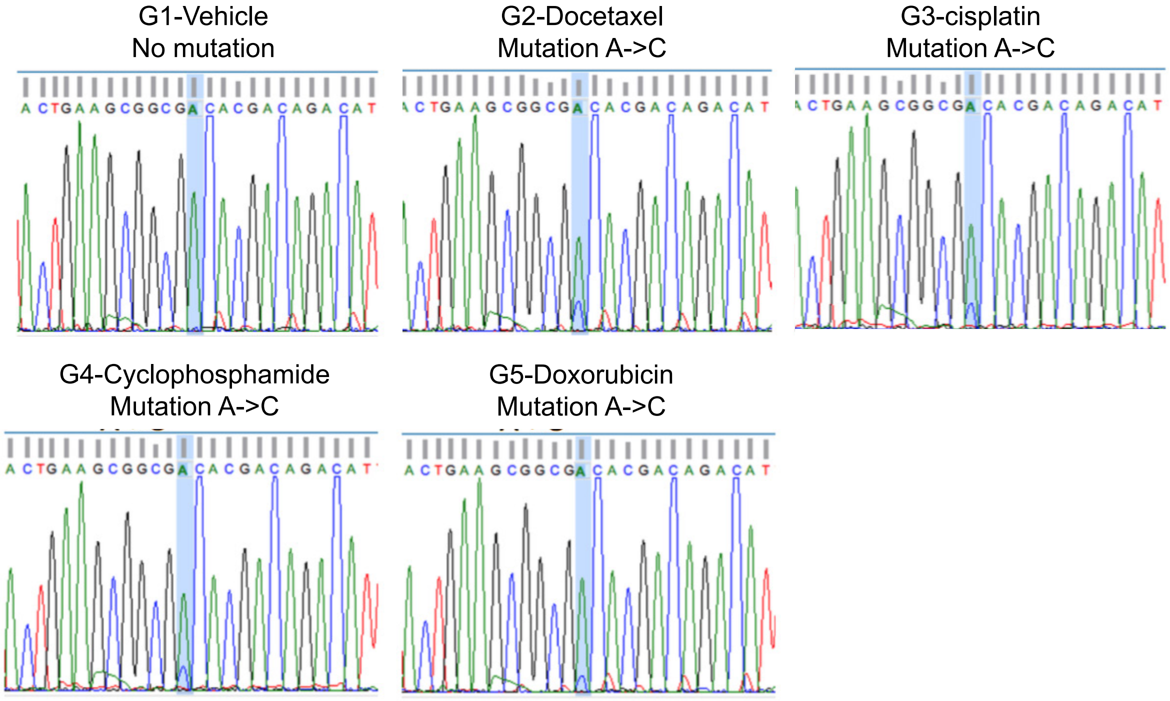

Figure S2. Sanger sequencing results across multiple samples for the ERBB4 gene. Sample G1 does not have the ERBB4 mutation, while Samples G2-G5 show low levels of it. These samples were obtained from residuals of the treated TM00099 tumors, though not the same samples used for the exome-seq.

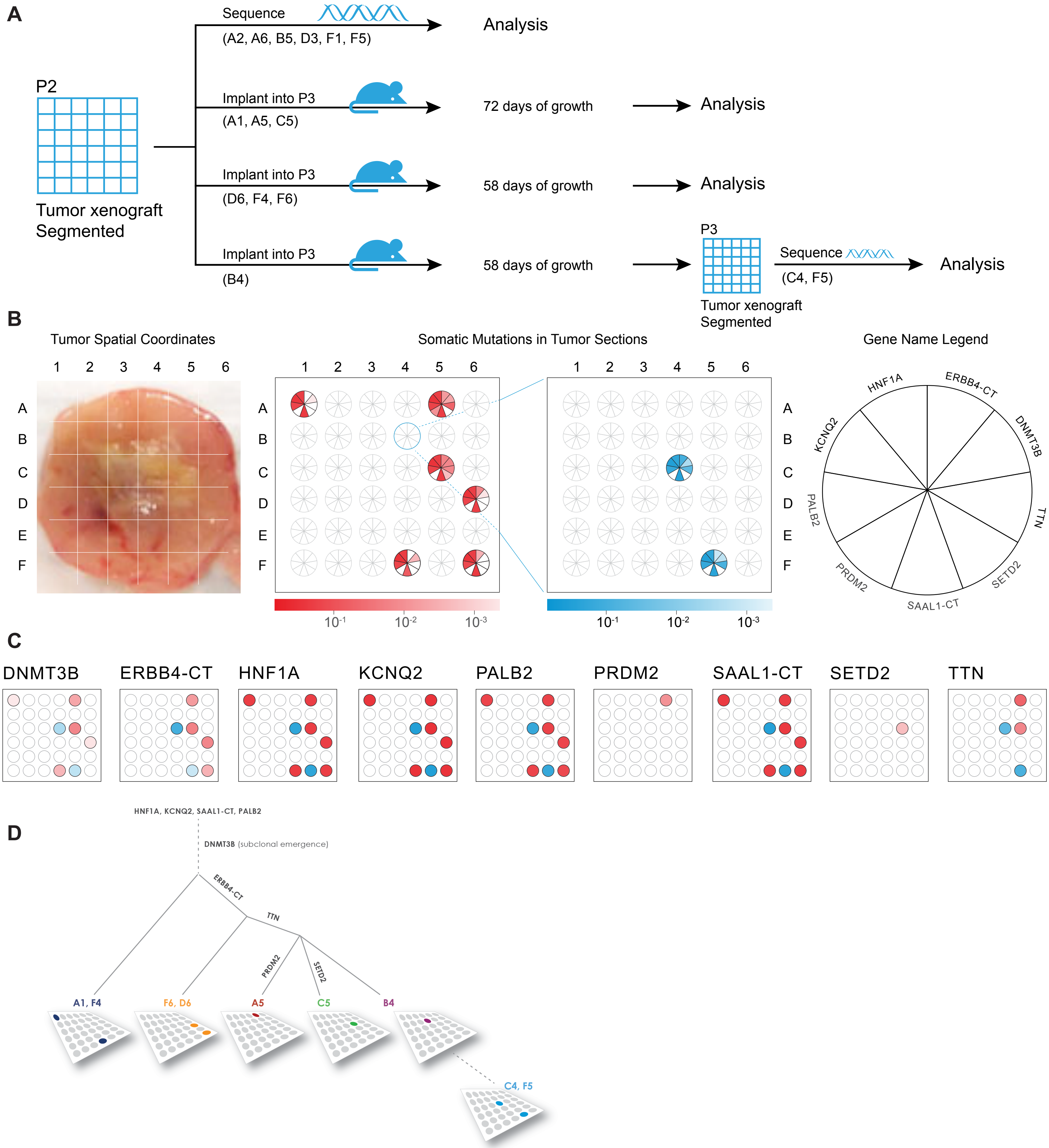

Figure S3. Spatiotemporal dissection of TM00096 tumor. (A) Fragments from the dissected P2 xenograft (location specified by rectilinear coordinates) were engrafted into P3 mice, grown, and sequenced. One P3 was dissected and two fragments (C4, F5) were sequenced (bottom row). (B) Visualization of ddPCR mutant AFs. Darker shading indicates higher AF, with mutations distinguished by wedge angle. (C) Alternate visualization with mutations in separate plots. (D) Inferred phylogenetic tree. HNF1A, KCNQ2, SAAL1-CT, and PALB2 have high AF in all samples, indicating truncal occurrence. DNMT3B, TTN, and ERBB4 are in multiple samples but often at low AF(<3%), indicating early but persistent subclonality.

	P0	P3	P4	control	cisplatin	cyclophosphamide	doxorubicin	docetaxel
**Allele Frequency**	0.6559	0.6387	0.5786	0.4883	0.5598	0.5089	0.5281	0.5211

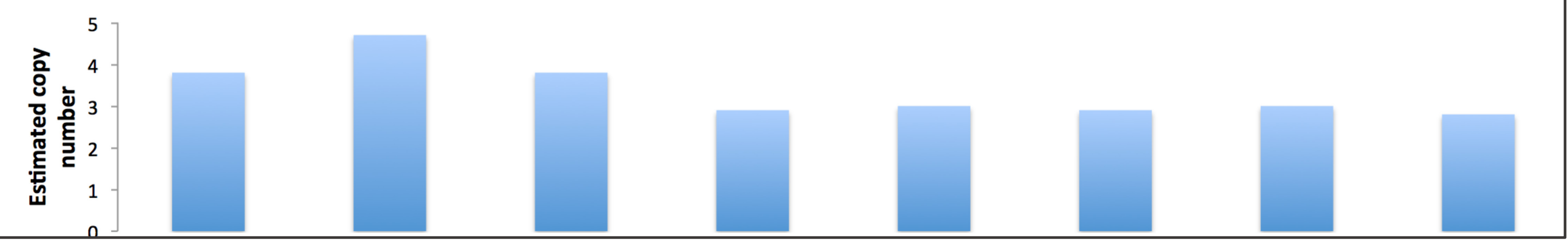

Figure S4. Exome-seq copy number uncertainties for TM00096. CONTRA-based CN estimates are plotted vs mutant AF at the MUC4 locus. Correlation of CN and AF suggests importance of CN to cellularity estimation. However, CN estimates vary substantially and do not correspond to integer values, limiting inference of subclone genotypes and cellularity levels.

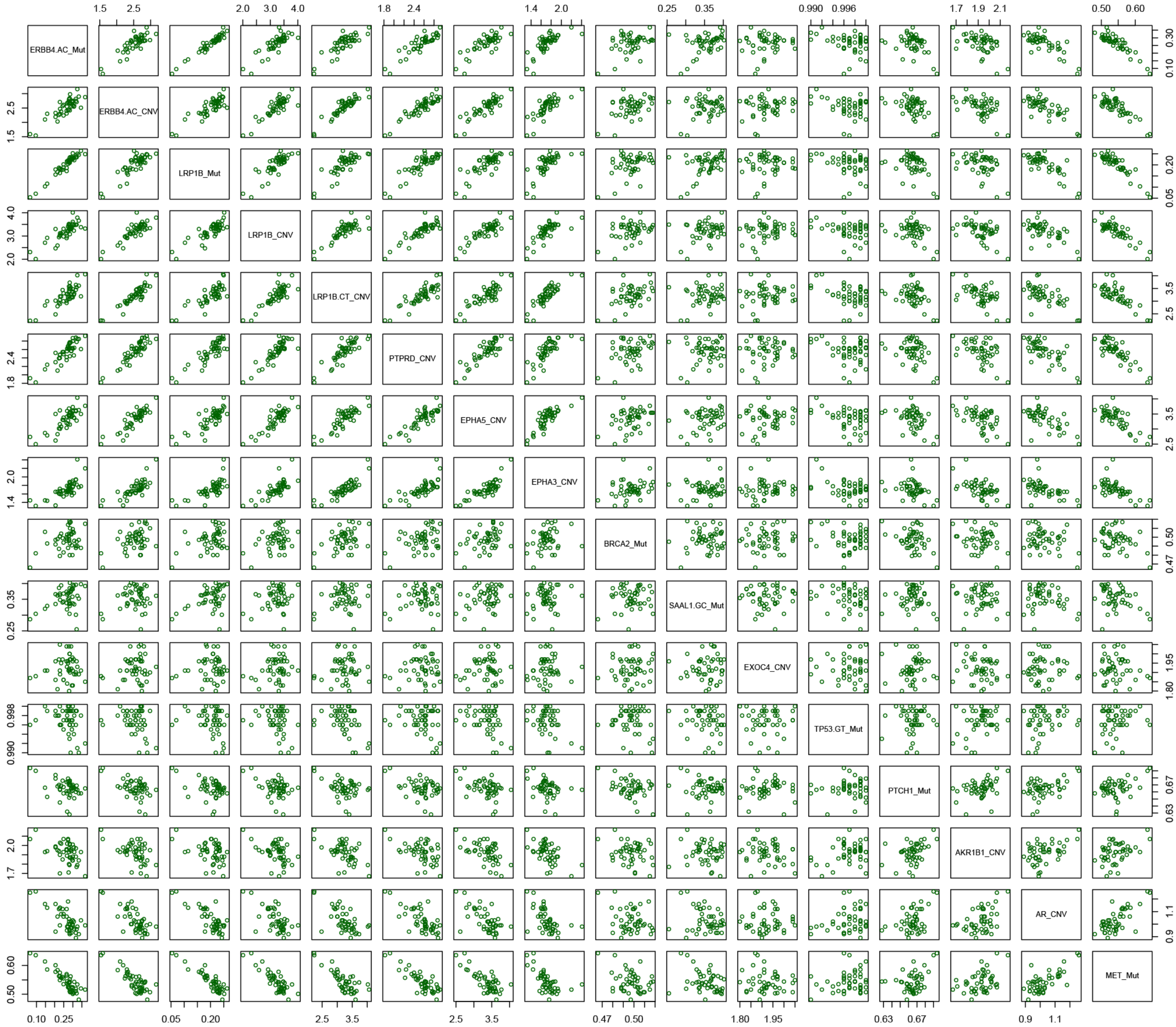

Figure S5. Pairwise comparisons of all mutant AFs and CNVs for the TM00099 tumor. Only samples that had sufficient DNA to samples to perform measurements at all loci are shown. Full data are in Table S1.

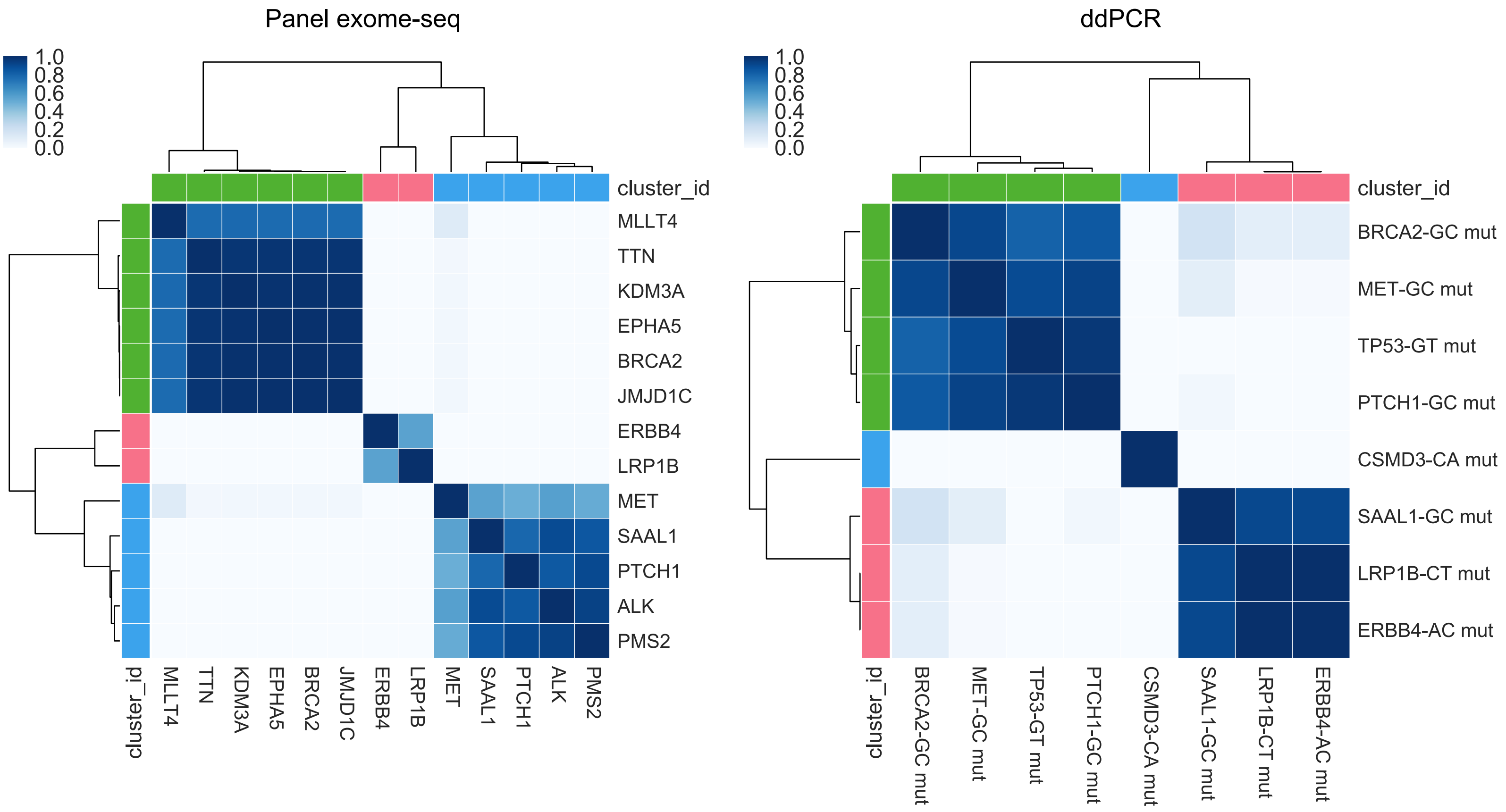

Figure S6. Comparison of Pyclone results for panel exome sequencing and ddPCR.
(left) The posterior similarity matrix generated by PyClone based on variant AF (allele frequency) and CN (copy number) estimates for all TM00099 samples as measured on the CTP exome panel. AFs of variant loci were computed by GATK, while copy numbers were estimated by CONTRA. Mutations called by the pipeline of Figure S1 are shown.
(right) The posterior similarity matrix generated by PyClone with variant AF and CN estimates for TM00099 samples for sites measured by ddPCR. The improved inference from ddPCR is apparent from the stronger bimodality in correlation values. Pyclone was run with option –prior total_copy_number and read counts were modeled using the pyclone_beta_binomial option.

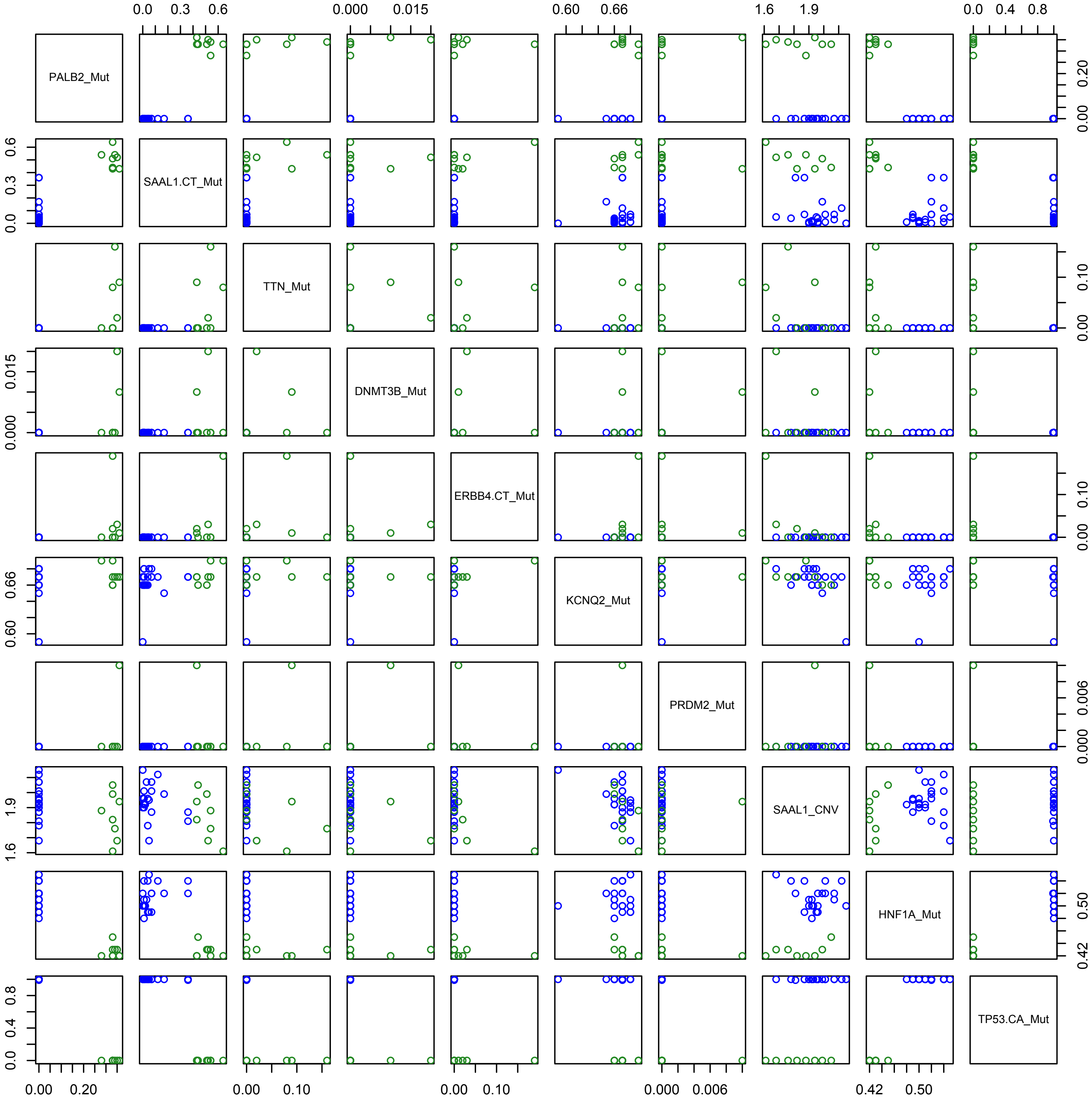

Figure S7. Pairwise comparisons of ddPCR measured quantities for TM00096. Green = spatiotemporal samples. Blue = treatment samples. The spatiotemporal and treated samples form two clusters because the spatiotemporal samples have a recent common ancestry.

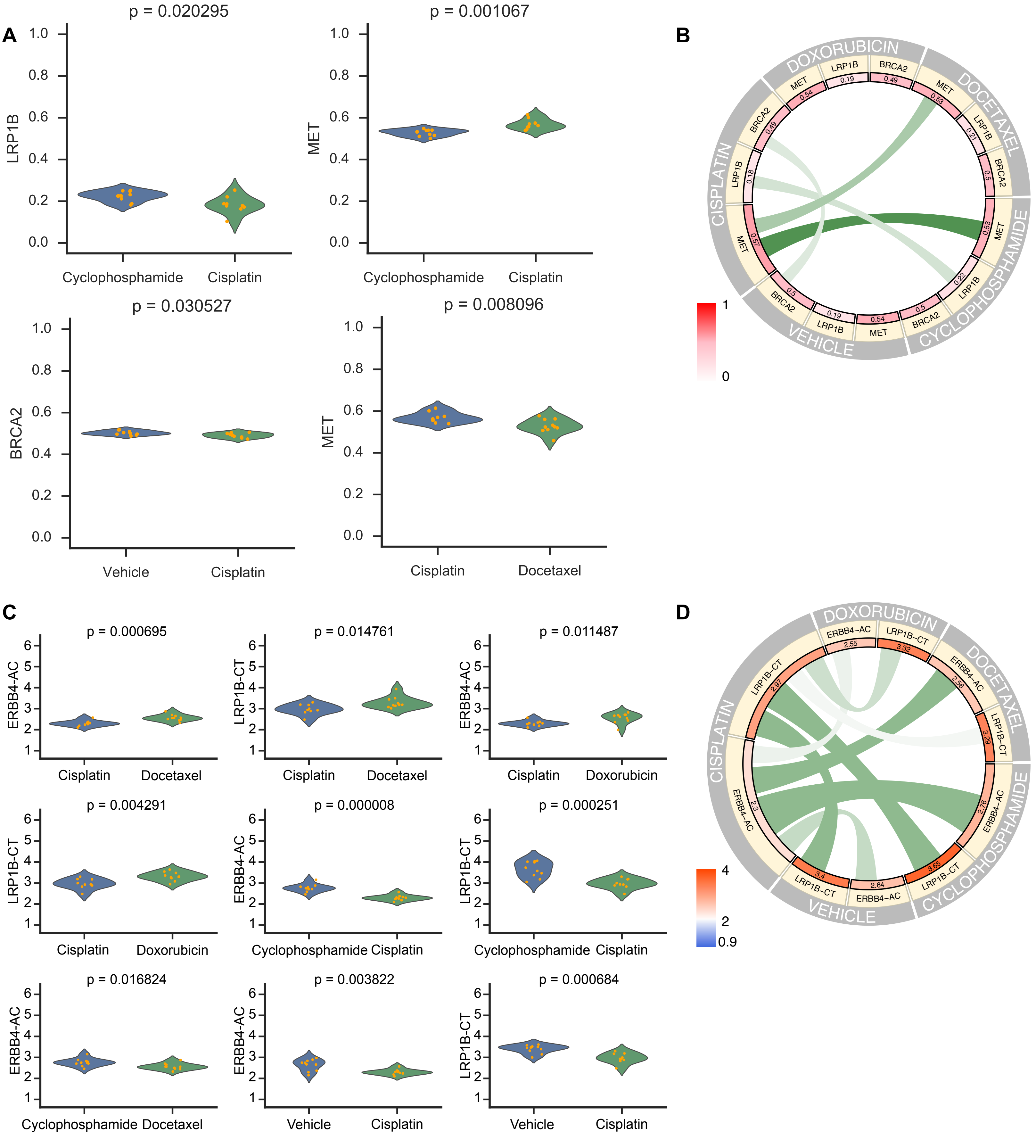

Figure S8. Comparisons of mutant AF and CN across single treatment cohorts.
(A) Significant mutant AF comparisons (Welch's test, alpha = 0.05, no multiple hypothesis correction).
(B) Chord diagram of cohorts with significant mutant AF differences. Line width indicates p-value. Average cohort AF is indicated under each gene and by pink scale.
(C) Significant CN comparisons (Welch's test, alpha = 0.05, Benjamini-Hochberg correction).
(D) Chord diagram showing significant CN differences.
